# PolDIP2 interacts with human PrimPol and enhances its DNA polymerase activities

**DOI:** 10.1093/nar/gkw175

**Published:** 2016-03-16

**Authors:** Thomas A. Guilliam, Laura J. Bailey, Nigel C. Brissett, Aidan J. Doherty

**Affiliations:** Genome Damage and Stability Centre, School of Life Sciences, University of Sussex, Brighton BN1 9RQ, UK

## Abstract

Translesion synthesis (TLS) employs specialized DNA polymerases to bypass replication fork stalling lesions. PrimPol was recently identified as a TLS primase and polymerase involved in DNA damage tolerance. Here, we identify a novel PrimPol binding partner, PolDIP2, and describe how it regulates PrimPol's enzymatic activities. PolDIP2 stimulates the polymerase activity of PrimPol, enhancing both its capacity to bind DNA and the processivity of the catalytic domain. In addition, PolDIP2 stimulates both the efficiency and error-free bypass of 8-oxo-7,8-dihydrodeoxyguanosine (8-oxoG) lesions by PrimPol. We show that PolDIP2 binds to PrimPol's catalytic domain and identify potential binding sites. Finally, we demonstrate that depletion of PolDIP2 in human cells causes a decrease in replication fork rates, similar to that observed in PrimPol^−/−^ cells. However, depletion of PolDIP2 in PrimPol^−/−^ cells does not produce a further decrease in replication fork rates. Together, these findings establish that PolDIP2 can regulate the TLS polymerase and primer extension activities of PrimPol, further enhancing our understanding of the roles of PolDIP2 and PrimPol in eukaryotic DNA damage tolerance.

## INTRODUCTION

In eukaryotes, the replicative polymerases (Pols) α, δ and ϵ are primarily responsible for bulk DNA replication. These enzymes, which duplicate DNA with extremely high efficiency and accuracy, are prone to stalling upon encountering helix-distorting DNA lesions generated by DNA damage (1). The inability of the replicative polymerases to synthesize across damaged nucleobases in turn causes replication fork stalling and requires DNA damage tolerance mechanisms in order to proceed with replication and prevent fork collapse (2,3).

A number of distinct replication restart mechanisms exist in order to permit continued replication in the presence of damage. These include the firing of dormant origins downstream of the damage, the generation of new Okazaki fragments on the lagging strand or repriming on the leading strand, the use of an alternative sister template to bypass the damage via homologous recombination, and direct synthesis past the damage through translesion synthesis (TLS) (2–4). Whilst it has been appreciated that specialized DNA polymerases, particularly those of the Y-family, play a key role in eukaryotic damage tolerance by TLS, the role of DNA primases in this process has until recently been mostly overlooked. However, novel roles for primases in DNA repair and damage tolerance are emerging from both prokarya and eukarya (5). Notably, archaeal replicative primases are now known to display TLS activity (6), whilst most eukaryotes possess a specialized primase-polymerase (PrimPol) that plays roles in TLS and re-priming (7).

PrimPol is a member of the archaeo-eukaryotic primase (AEP) superfamily (5) and demonstrates primer synthesis capabilities with both nucleoside and deoxynucleoside triphosphates (NTPs and dNTPs) (8–10). In addition, the enzyme displays robust template-dependent TLS polymerase activity, which it utilizes to bypass pyrimidine 6-4 pyrimidone photoproducts (6-4PPs) and 8-oxo-7,8-dihydrodeoxyguanosine (8-oxoG) lesions (8,9). These activities have been shown to be relevant *in vivo* as cells lacking PrimPol show increased sensitivity to DNA damaging agents and decreased replication fork speeds (8,11). *In vivo* PrimPol localizes to both the nucleus and mitochondria, indeed PrimPol^−/−^ cells also present mitochondrial DNA (mtDNA) replication defects (9,12). Unlike canonical Y-family polymerases, PrimPol does not seem to be regulated through interactions with PCNA (13). Despite this, PrimPol is a low fidelity polymerase and alternative mechanisms must exist to regulate its activity *in vivo* (13). One such regulator is the inherent distributive nature of the enzyme, which limits incorporation to ∼4 nucleotides per binding event (11). In addition, PrimPol's activities are also regulated by its association with single-strand binding proteins (SSBs) (13). Interactions with these proteins may also be involved in the recruitment of PrimPol to the replisome (14). Nevertheless, it is likely that additional replication factors also regulate the activity of PrimPol during replication.

In addition to SSBs, polymerase δ-interacting protein 2 (PolDIP2 or PDIP38) was also identified in a pull-down screen as a possible cellular binding partner of PrimPol (13). Recently, it was reported that PolDIP2 may play a role in DNA damage tolerance, specifically through the regulation of TLS (15,16). However, PolDIP2 is a relatively understudied protein, which has been ascribed multiple roles *in vivo* and its function in DNA replication is still unclear. This protein was first identified through yeast two-hybrid screening as a binding partner of the p50 subunit of Pol δ, as well as PCNA (17). Further characterisation suggested that PolDIP2 is a mitochondrial protein (18), which inhibits Pol δ and might be involved in Pol δ-mediated viral DNA replication (19). However, in contrast to this initial characterization, more recent studies have identified that PolDIP2 also localizes to the nucleus (20) and actually stimulates the activity of Pol δ *in vitro* (16). Additionally, PolDIP2 has been shown to increase the processivity and fidelity of lesion bypass by Pols λ and η (16). In addition, the protein was previously found to interact with Pols η, ζ, and Rev1, with depletion causing persistent Pol η nuclear foci and decreased cell survival following UV damage (15).

Aside from a potential role in DNA replication, PolDIP2 has also been reported to have roles in regulating the nuclear redox environment (21), mitotic spindle formation (22), and in pre-mRNA processing in the spliceosome (20). The multitude of roles assigned to PolDIP2 highlights the multi-functional nature of the protein but also obscures the interpretation of many results. This has brought into question the role of PolDIP2 in TLS and DNA replication (20) thus necessitating further study to properly characterize its function in these areas.

In this report, we aimed to further explore the regulation of PrimPol, and the role of PolDIP2 in TLS, by investigating the relationship between these two proteins. We observed that PolDIP2 stimulates the polymerase activity of PrimPol. This stimulation appears to be the result of an increase in DNA binding by PrimPol in the presence of PolDIP2, consequently producing an increase in the processivity of the enzyme to levels not previously observed. Furthermore, we found that PolDIP2 alone is sufficient to stimulate the efficiency and fidelity of 8-oxoG bypass by PrimPol, an effect similar to that seen with Pols λ and η in the presence of PCNA, RPA and PolDIP2 (16). We used cross-linking and mass spectrometry (MS) analysis to investigate the interaction between PrimPol and PolDIP2. We found that PolDIP2 binds to the catalytic domain of PrimPol and identify potential binding sites, including a region displaying homology to the previously identified PolDIP2-interacting region of Pol η (15). Finally, we explored the role of PolDIP2 in replication *in vivo*. We observed that depletion of PolDIP2 decreased replication fork rates in human cells following UV irradiation. The level of decrease in replication fork rates was similar to that observed in the absence of PrimPol and, additionally, no further decrease in fork speeds was evident when PolDIP2 was depleted in PrimPol^−/-^ cells. Together, these findings support a role for PolDIP2 in regulating TLS and enhancing the primer extension activities of PrimPol during DNA replication. We propose that PolDIP2 acts specifically to enhance PrimPol's primer extension and TLS activities, whilst having minimal effect on its priming function. Overall, this study provides further evidence for the involvement of both PrimPol and PolDIP2 in TLS during DNA replication in higher eukaryotes.

## MATERIALS AND METHODS

### Expression and purification of recombinant proteins

Full-length Human PrimPol and PrimPol^24–354^ were cloned and purified as described previously (11,13). Recombinant GST-PolDIP2, Pol η, PCNA, RPA, and mtSSB, were expressed and purified as previously reported (16,23–25). Untagged PolDIP2 was purified from GST-PolDIP2 through cleavage of the GST tag using PreScission protease before further purification using a GSTrap column and ion exchange chromatography to remove the cleaved GST tag and protease.

PolDIP2^51–368^ was constructed by PCR using the following forward and reverse primers;

FWD: 5′-GTTTCTTCATATGCTCTCGTCCCGAAACCGACCAGAGGGCAAA-3′, REV: 5′-CAAAGAAGCGGCCGCCTACCAGTGAAGGCCTGAGGG-3′, followed by cloning into pET28a via the introduced NdeI and NotI restriction sites. The resulting construct was expressed in *E. coli* at 20°C overnight. Cells were then pelleted before resuspension in buffer A (50 mM Tris–HCl (pH 7.5), 200 mM NaCl, 30 mM imidazole, 10% (v/v) glycerol, 17 μg/ml PMSF, 34 μg/ml benzamidine) supplemented with IGEPAL to a final concentration of 0.5%. Cells were lysed by sonication and the lysate clarified by centrifugation. The clarified lysate was applied to a Ni^2+^-NTA agarose chromatography column (Qiagen) pre-equilibrated with buffer A. The protein was eluted with buffer A supplemented with 300 mM imidazole following sufficient washing. The resulting eluate was diluted into buffer B (50 mM Tris–HCl (pH 8.5), 50 mM NaCl, and 10% (v/v) glycerol) and subject to ion exchange chromatography using a 5 ml MonoS column (GE Healthcare) prior to a gradient elution with buffer B containing 1 M NaCl. Fractions containing PolDIP2^51–368^ were further purified by size exclusion chromatography on a Superdex S-75 analytical gel-filtration column (GE Healthcare) equilibrated with buffer C (50 mM Tris–HCl (pH 7.5), 300 mM NaCl, 10% (v/v) glycerol).

Following purification, all proteins were snap frozen in liquid nitrogen and stored at −80°C. Protein concentrations were calculated based on sample absorbance at 280 nm and corrected to the protein specific extinction coefficient as determined using the ProtParam tool (ExPASy).

### Electrophoretic mobility shift assays

Assays were performed as previously described (13) in 20μl reactions containing 10 mM Bis–Tris–propane–HCl (pH 7.0), 10 mM MgCl_2_, 100 μM DTT, 20 nM primer/template substrate (sequences 2 and 6, Supplementary Table S1), and varying concentrations of PrimPol and PolDIP2 (as indicated on individual figures). Reactions were resolved on 5% (v/v) native polyacrylamide gels at 75 V for 60 min in 0.5× TBE buffer. Fluorescently labeled DNA was detected using a FujiFilm FLA-5100 image reader.

### DNA primase assays

Reactions were assembled in buffer containing 10 mM Bis–Tris–propane–HCl (pH 7.0), 10 mM MgCl_2_, and 100 μM DTT, supplemented with 2 μM PrimPol, 250 μM dNTPs, 2.5 μM FAM dNTPs (dATP, dCTP, dUTP) (Jena-Biosciences), 1 μM single-stranded template (sequence 7, Supplementary Table S1), and varying concentrations of PolDIP2 or GST (as indicated on individual figures). Reactions were incubated at 37°C for 15 min before quenching in binding-washing (B-W) buffer (10 mM Tris–HCl pH 7.5, 500 mM NaCl, 10 mM EDTA). Quenched reactions were incubated with ∼20 μl streptavidin coated beads for 1 h at 4°C to allow capture of the DNA templates and primase reaction products. Following capture, beads were washed three times with 1 ml volumes of B-W buffer before final suspension in 20 μl stop buffer (95% formamide solution with 0.25% bromophenol blue and xylene cyanol dyes). Samples were then boiled for 5 min and products detected by resolution on a 15% (v/v) polyacrylamide gel containing 7M urea and 1× TBE buffer run at 850 V for 2.5 h in 1× TBE. Reaction products were visualized using a FujiFilm FLA-5100 image reader.

### DNA primer extension assays

Reactions (20 μl) were assembled containing; 20 nM 5′ hexa-chlorofluorescein (HEX)-labeled DNA primers annealed to the appropriate DNA templates (Supplementary Table S1), 10 mM Bis–Tris–propane–HCl (pH 7.0), 10 mM MgCl_2_, 100 μM DTT, and 100 μM dNTPs. Reactions were supplemented with varying amounts of PrimPol or Pol η (as indicated in figure legends), and incubated at 37°C (time points are shown in figure legends). Where present, accessory proteins were added prior to the addition of the enzyme at the concentrations indicated on figures. In the case of single nucleotide incorporation analysis, dNTPs were substituted for 100 μM of the single dNTP being analysed (dATP, dCTP, dGTP or dTTP). Reactions were quenched with buffer containing 95% formamide, 0.05% bromophenol blue, 0.09% xylene cyanol and 200 nM competitor oligonucleotide. Quenched reactions were heated to 95°C for 5 min before electrophoresis on a 15% (v/v) polyacrylamide/ 7 M urea gel. Extended fluorescent primers were imaged using a FujiFilm FLA-5100 image reader. All quantification was performed using ImageQuant TL software (GE Life Sciences). Data were plotted and analysed using GraphPad Prism 6.

### Polymerase processivity assays

PrimPol's processivity in the presence of varying amounts of PolDIP2 was analysed using the method previously described (11). Extension reactions were assembled containing 100 nM PrimPol, varying concentrations of PolDIP2, 40 nM primer/template substrate (sequences 2 and 6, Supplementary Table S1), 10 mM Bis–Tris–propane–HCl (pH 7.0), 10 mM MgCl_2_, and 100 μM DTT, and incubated at 37°C. Reactions were initiated by supplementation with 100 μM dNTPs and 1 mg/ml sonicated herring sperm trap DNA. Reaction products were monitored over a time course and quenched at various time points (as indicated in figure legends) using buffer containing 95% formamide, 0.05% bromophenol blue, 0.09% xylene cyanol and 200 nM competitor oligonucleotide. The efficiency of the trap DNA was analysed using control reactions containing the trap DNA in the initial reaction assembly to ensure no extension was observed. Extension products were resolved and imaged as described in ‘DNA primer extension assays’. Reaction products were quantified using ImageQuant TL software (GE Life Sciences) and the previously described method (11).

### Crosslinking and mass spectrometry analysis

Purified untagged-PolDIP2 and PrimPol^24–354^ were mixed at equimolar concentrations in buffer containing 50 mM HEPES (pH 7.5), 150 mM NaCl, and 10% (v/v) glycerol, for 30 min on ice to allow binding. Following this, protein samples were supplemented with bis(sulfosuccinimidyl)suberate (BS^3^) crosslinker at increasing concentrations (from 1:1 to 20:1 crosslinker:protein molar ratios). Samples were incubated on ice for 45 min to 1 h to allow crosslinking reactions to proceed, before quenching with 50 mM Tris and further incubation for 15 min. Crosslinked samples were supplemented with Laemmli buffer and resolved by electrophoresis on a polyacrylamide gel. Bands corresponding to a 1:1 PrimPol:PolDIP2 complex molecular weight were excised and processed for mass spectrometry (MS) as described (26).

MS samples were analysed using a nano-LC–MS (ThermoFisher U3000 nanoLC and Orbitrap XL mass spectrometer) as previously described (27). Raw MS and MS/MS spectra were converted to the .mgf format using Compass (28) and searched against the SwissProt database with Mascot (Matrix Science). Search parameters employed a precursor tolerance of 5 ppm and a fragment ion tolerance of 0.6 Da. Crosslinked peptides were identified by analysing .mgf files using StavroX crosslinking analysis software as described previously (29).

### Cell culture and DNA fibre analysis

MRC5 cells were cultured in MEM supplemented with 15% FCS, 1% l-glutamine and 1% PenStrep (v/v), at 37°C in CO_2_ controlled incubators. Cells were transfected with PolDIP2 siRNA (SMARTpool ON-TARGET plus siRNA Thermo Fisher Scientific), or mock siRNA treated, using Lipofectamine RNAiMAX (Invitrogen) according to the manufacturer's instructions. 72 h following siRNA transfection cells were subject to DNA fibre analysis as described previously (8). All DNA fibre analysis was performed in triplicate. MRC5 PrimPol^−/−^ cells were generated using the zinc finger nuclease knockout method according to the manufacturer's recommendations (Sigma-Aldrich).

## RESULTS

### PolDIP2 stimulates the polymerase activity of PrimPol

**Figure 1.**
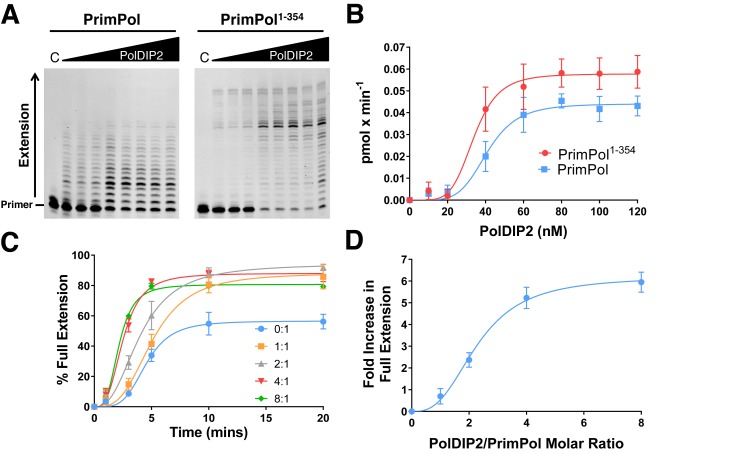
PolDIP2 stimulates the polymerase activity of PrimPol. (**A**) PrimPol or PrimPol^1–354^ (20 nM) were incubated with 5′ labeled primer/template DNA substrates (20/50-mer; 20 nM) and dNTPs (100 μM) in the presence of increasing concentrations of GST-PolDIP2 (0, 10, 20, 40, 60, 80, 100, and 120 nM) for a single 5 min timepoint. ‘C’ indicates the no enzyme control reaction. (**B**) Relative increase in the rate of primer extension by PrimPol and PrimPol^1–354^ in the presence of increasing GST-PolDIP2 concentrations (0, 10, 20, 40, 60, 80, 100 and 120 nM). Data were fitted using GraphPad Prism 6 software. Values are the means of four independent experiments. Error bars are ±SD. (**C**) PrimPol generates a greater proportion of fully extended primers in the presence of PolDIP2. PrimPol (100 nM) was incubated with 5′ labelled primer/template DNA substrates (20/50-mer; 20 nM) and dNTPs (100 μM) in the presence of increasing GST-PolDIP2 concentrations (0, 0.1, 0.2, 0.4 and 0.8 μM) for increasing timepoints (0, 1, 3, 5, 10 and 20 mins) Fully extended primers (as indicated on Supplementary Figure S1) were quantified for each timepoint as a percentage with respect to the total primers present. Data were fitted using GraphPad Prism 6 software. Values are the means of three independent experiments. Error bars are ± SD. Representative gels used for quantification are shown in Supplementary Figure S1. (**D**) Fold increase in fully extended primers by PrimPol in the presence of increasing PolDIP2/PrimPol molar ratios at a single 3 min timepoint. Data were fitted using GraphPad Prism 6 software. Values are the means of three independent experiments. Error bars are ±SD. Representative gels used for quantification are shown in Supplementary Figure S1.

PolDIP2 was originally identified in a large-scale pull-down mass spectroscopy screen previously performed to identify cellular binding partners of PrimPol (13,30). This screen also identified the single-strand binding proteins (SSBs), RPA and mtSSB as interacting partners of PrimPol, whilst an interaction with PCNA was not established. Further studies suggested that RPA and mtSSB regulate PrimPol's enzymatic activities and revealed that, unlike canonical TLS polymerases, PrimPol is not stimulated by the presence of PCNA *in vitro* (13). In light of reports implicating PolDIP2 in the regulation of TLS (15,16), we aimed to analyze whether PolDIP2 might also act as a PrimPol regulator. To do this, we employed primer extension assays on a 20/50-mer DNA primer/template substrate (sequences 2 and 4, Supplementary Table S1), in the presence of increasing concentrations of GST-PolDIP2. When titrated into primer extension reactions containing PrimPol, we observed that PolDIP2 stimulated the activity of the enzyme, producing an increase in the amount of extended primers in a dose-dependent manner (Figure 1A). Notably, PolDIP2 generated a similar effect when titrated into reactions containing PrimPol^1–354^, a truncation of the enzyme comprising the catalytic AEP domain only. Intriguingly, when plotted, these data produced a sigmoidal kinetic profile, suggesting that PrimPol may bind multiple PolDIP2 molecules (Figure 1B). Indeed, assays using both full-length PrimPol and PrimPol^1–354^, generated Hill coefficients of 5.176 ± 1.481 and 5.258 ± 1.466 respectively, revealing positive cooperativity in PolDIP2 binding. The *K*_half_ values for both PrimPol and PrimPol^1–354^ were 41 ± 1.817 nM and 33.93 ± 2.112 nM, respectively, with stimulation slightly more pronounced for the truncated enzyme.

Furthermore, at higher PrimPol and PolDIP2 concentrations an increase in the length of extended primers, with a significant increase in the amount of fully extended primers, was observed (Figure 1C and D, and Supplementary Figure S1). Notably, the GST-tag was not responsible for this stimulation as GST alone did not effect the polymerase activity of PrimPol (Supplementary Figure S1). In line with previous reports (16), GST-PolDIP2 was used for these assays due to the ease of purification and increased solubility over the untagged protein. These results establish that PolDIP2 is able to stimulate the polymerase activity of PrimPol, increasing both the amount and length of extended primers.

Given the stimulatory effects of PolDIP2 on the polymerase activity of PrimPol, we next sought to assess if it also modulated PrimPol's primase activity. To determine this, we analysed the primase activity of the enzyme on a 66-mer mixed sequence ssDNA template (sequence 7, Supplementary Table S1) in the presence of increasing concentrations of GST-PolDIP2 or GST alone. As observed previously (31), PrimPol was able to synthesize primers on this ssDNA template in the absence of PolDIP2 or GST. When present, PolDIP2 did not significantly increase the amount of primers synthesized (Supplementary Figure S2). However, in the presence of PolDIP2, PrimPol did appear to extend generated primers further. This supports a scenario where PolDIP2 is unable to increase the rate at which primers are synthesized but is able to increase the rate and length to which these primers are extended.

### PolDIP2 enhances PrimPol's DNA binding

PrimPol has previously been shown to bind relatively poorly to DNA (11), thus it seems likely that additional factors assist it in the coordination of DNA *in vivo*. Previously, it was reported that PolDIP2 increases the DNA binding affinity of Pol λ, whilst lacking the capacity to bind DNA itself (16). Consequently, the effect of PolDIP2 on the DNA binding of PrimPol was analysed. To this end, electrophoretic mobility shift assays (EMSAs) were performed using a 20/97-mer DNA primer/template substrate (sequences 2 and 6, Supplementary Table S1) in the presence of varying amounts of untagged-PolDIP2 and PrimPol. In order to analyse the effect of PolDIP2 on the DNA binding affinity of the catalytic domain of PrimPol only, a truncation of the enzyme (PrimPol^24–354^) was used that contained only the AEP domain. Importantly, this eliminates possible binding artefacts being introduced by the presence of the ssDNA-binding zinc finger domain (11).

**Figure 2.**
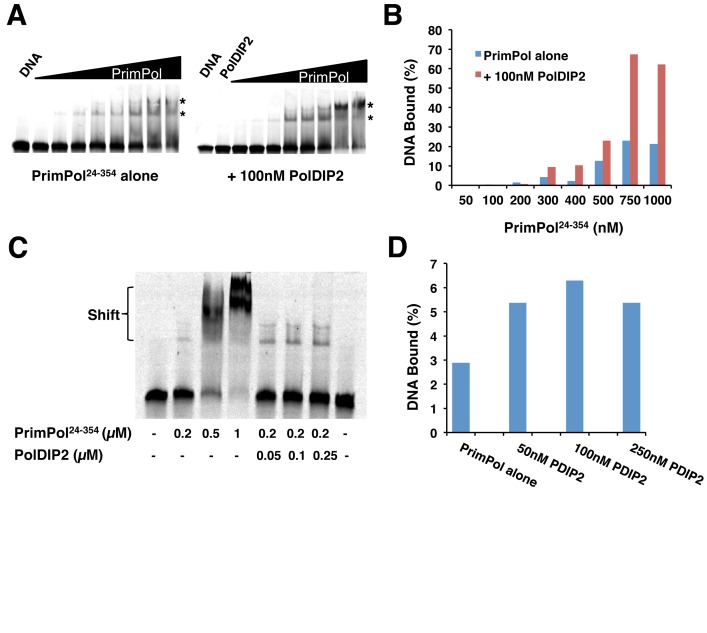
PolDIP2 enhances the DNA binding affinity of the catalytic domain of PrimPol. (**A**) Increasing concentrations of PrimPol^24–354^ (0.05, 0.1, 0.2, 0.3, 0.4, 0.5, 0.75, 1 μM) were incubated in EMSA reactions containing 5′-labeled primer/template substrates (20/97-mer) in the absence or presence of untagged-PolDIP2 (100 nM). The ‘PolDIP2’ lane indicates the no PrimPol control, showing that PolDIP2 (100 nM) alone does not bind to the primer/template substrate. The asterisk denotes a super-shifted complex. Data are representative of three repeat experiments. (**B**) Quantification of the data presented in A. For each PrimPol concentration the percentage of DNA bound (in relation to the total DNA) was calculated and compared for EMSAs containing PrimPol only, or PrimPol and PolDIP2. (**C**) PrimPol^24–354^ alone (0, 0.2, 0.5 and 1 μM) and with increasing concentrations of PolDIP2 (200 nM PrimPol; 0, 50, 100 and 250 nM PolDIP2) was incubated in EMSA reactions containing 5′-labeled primer/template (20/97-mer) substrates. (**D**) Quantification of the data shown in ‘C’. For each PolDIP2 concentration the percentage of DNA bound (in relation to the total DNA) was calculated. Reactions containing PolDIP2 only again confirm that PolDIP2 alone does not bind to the primer/template substrate.

When titrated into EMSAs supplemented with a fixed concentration of PolDIP2, PrimPol bound to a significantly increased amount of DNA compared to EMSAs with PrimPol alone (Figure 2A and B). Consistent with previous reports, PolDIP2 alone showed no ability to bind the DNA substrate (16), suggesting that this increase in binding was due to PolDIP2's effect on PrimPol. A similar effect was also observed when PolDIP2 was titrated into EMSAs with a fixed concentration of PrimPol (Figure 2C and D). Together, these data show that PolDIP2 exerts a similar influence on the DNA binding avidity of PrimPol, as previously reported for Pol λ (16). Furthermore, this confirms that PrimPol forms a complex with PolDIP2 on the DNA. As these assays were conducted with the AEP domain alone, it suggests that PolDIP2 binds PrimPol via the catalytic domain. This is in agreement with the stimulation observed in primer extension assays, as is also the case with Pol λ (16).

### PolDIP2 increases the processivity of PrimPol

PrimPol is a poorly processive polymerase, incorporating only ∼4 nucleotides per binding event (11). Interestingly, it has previously been shown that this low processivity is partially due to the restraining effect of the zinc finger (ZnF) domain, in combination with the enzyme's weak affinity for DNA (11). The ssDNA binding activity of the ZnF domain is thought to produce inter-domain collisions with the catalytic AEP domain following synthesis of ∼4 nucleotides, thus limiting PrimPol's contribution to DNA synthesis to very short stretches. However, in light of the fact that PolDIP2 can increase PrimPol's DNA binding ability, we hypothesized that the protein may also increase PrimPol's processivity. To investigate this, we employed a standard primer extension assay on DNA primer/template substrates (20/97-mer) in the presence of excess unlabelled trap DNA. Pre-incubation of PrimPol and DNA template before initiation with dNTPs and a DNA trap allowed incorporations during a single association/dissociation event to be analysed and thus enabled us to determine the processivity of the enzyme.

**Figure 3.**
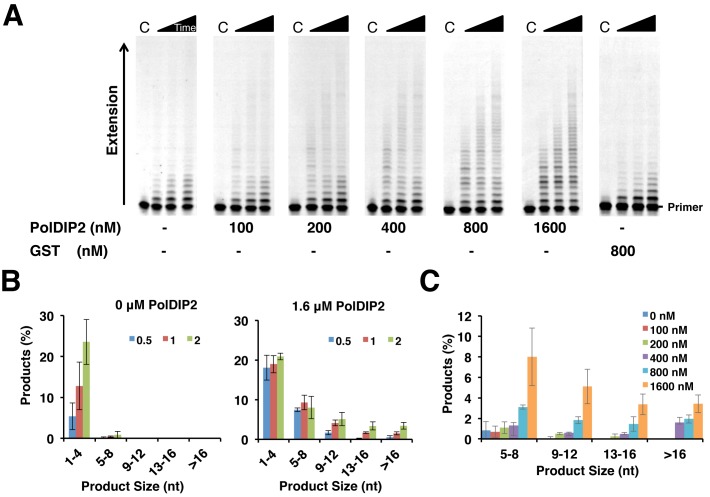
PolDIP2 enhances the processivity of PrimPol. (**A**) PolDIP2 was titrated into reactions containing PrimPol (100 nM) and 5′ labeled primer/template substrates (20/97-mer; 20 nM). Reactions were initiated with dNTPs (100 μM) and excess trap DNA and quenched at 0.5, 1 and 2 min time-points. Reactions containing GST only show no increase in PrimPol's processivity. ‘C’ indicates the control reaction with trap DNA added before the enzyme. (**B**) Quantification of processivity reactions containing either PrimPol alone or PrimPol and PolDIP2 (1.6 μM). Reaction products were quantified as a function of their size in relation to the total primers present for each time-point. Data represent the means of three independent experiments. Error bars are ±SD. (**C**) Quantification of PrimPol processivity in the presence of increasing GST-PolDIP2 concentrations (as shown) at the 2 min timepoint. Reaction products were quantified as a function of their size in relation to the total primers present for each time-point. Data represent the means of three independent experiments. Error bars are ±SD.

In the absence of PolDIP2, PrimPol's processivity was in line with the previously determined levels, confirming the efficiency of the DNA trap (11). However, when titrated in identical reaction conditions, PolDIP2 produced a significant dosage-dependent increase in the processivity of PrimPol (Figure 3A–C). At the highest concentration of PolDIP2 (1.6 μM) and longest time point (2 min), products of more than 16 nucleotides in length were visible, representing a >4-fold increase in PrimPol's processivity. In addition to the increased length of the synthesized products, PrimPol also produced longer products more rapidly in the presence of PolDIP2. This is evidenced by analysing reaction products from the shortest time point where, in the absence of PolDIP2, PrimPol had still not synthesized products of four nucleotides in length. In contrast, in the presence of PolDIP2 products >8 nucleotides in length were apparent. Again, no stimulation of processivity was observed in the presence of GST alone, confirming that the GST tag is not causing this effect. Furthermore, untagged PolDIP2 was able to produce similar increases in processivity when used at higher concentrations (Supplementary Figure S3). Higher concentrations were probably required due to the decreased solubility of the protein in the absence of the GST tag. Together, these data reveal that in the presence of PolDIP2, PrimPol produces longer products more efficiently in a single binding event compared to PrimPol alone. Importantly, these results suggest that PrimPol is potentially involved in synthesis of longer stretches of DNA than previously thought.

### PolDIP2 does not allow PrimPol to displace SSBs

We previously observed that, unlike many replicative polymerases, PrimPol was unable to displace both RPA and mtSSB from DNA during synthesis and we proposed a model whereby these SSBs regulate PrimPol's activity to restrict the enzyme's potentially mutagenic contribution to DNA replication (13). However, in light of the increased processivity and DNA binding potential of PrimPol when in complex with PolDIP2, we postulated that this complex might be able to overcome negative regulation by SSBs. To test this hypothesis, we employed standard primer extension assays with PrimPol in the presence of PolDIP2 and RPA or mtSSB. Here, PolDIP2 was unable to relieve the inhibitory effects of RPA and mtSSB on the primer extension activity of PrimPol (Supplementary Figure S4). In each case, primer extension was significantly inhibited when compared to reactions in the absence of accessory proteins. These results show that even in the presence of PolDIP2, PrimPol is unable to displace SSBs from DNA and is therefore unable to overcome their negative regulatory effects.

### PrimPol is inhibited in presence of both PolDIP2 and PCNA

PolDIP2 has previously been found to interact with PCNA (17). Coupled with this, it has been shown that the protein can increase the affinity of Pol δ for PCNA, resulting in increased stimulation of the enzyme's polymerase activity in presence of PolDIP2 and PCNA over either factor alone (16). These studies suggest that PolDIP2 is able to act as a bridging factor to help tether polymerases to PCNA, leading to further stimulation of their activity. It was previously found that PrimPol does not interact with, and is not stimulated in the presence of, PCNA (13). This apparent lack of interaction and stimulation by PCNA is in contrast with canonical TLS polymerases, leading to speculation that PrimPol is not regulated by the PCNA-mediated polymerase switch mechanism, which regulates the activity of the Y-family TLS polymerases. However, given that PolDIP2 can interact with PCNA, it is possible that the protein might act as a bridging partner between PrimPol and PCNA, thus allowing PrimPol to be regulated by the classical PCNA-mediated polymerase switch model. To test this, we again used standard primer extension assays with PrimPol in the presence of PCNA alone or PCNA and PolDIP2. As shown previously, in the presence of PCNA PrimPol's activity is not affected, with no stimulation or inhibition observed compared to PrimPol alone (Supplementary Figure S5). However, somewhat unexpectedly, in the presence of both PCNA and PolDIP2, PrimPol's polymerase activity was actually inhibited when compared to reactions with the enzyme alone, or with PCNA only. This inhibitory effect may be due to PolDIP2 associating with, and stabilising, PCNA on the primer/template substrate and in turn blocking access by PrimPol. Nevertheless, this suggests that PolDIP2 does not act to tether PrimPol to PCNA, and in opposition to what has previously been reported with Pol δ, PCNA prevents stimulation of PrimPol by PolDIP2. These results further support the proposal that PrimPol is regulated by a mechanism distinct from that employed by canonical Y-family TLS polymerases.

### PolDIP2 increases the efficiency and fidelity of 8-oxoG bypass by PrimPol

PrimPol has previously been shown to possess TLS polymerase activity, displaying an ability to tolerate templating 8-oxoG lesions and 6–4PPs (8,9,11). It has been postulated that the ability of PrimPol to bypass 8-oxoG lesions may be of particular importance in the mitochondria given the localisation of PrimPol there and the fact that Pol γ deals poorly with these lesions (9,12). Intriguingly, PolDIP2 has also been shown to localize to the mitochondria (18,19), in addition to stimulating 8-oxoG bypass by Pols λ and η (16). Therefore, we set out to analyse the influence of PolDIP2 on the activity of PrimPol during 8-oxoG bypass.

**Figure 4.**
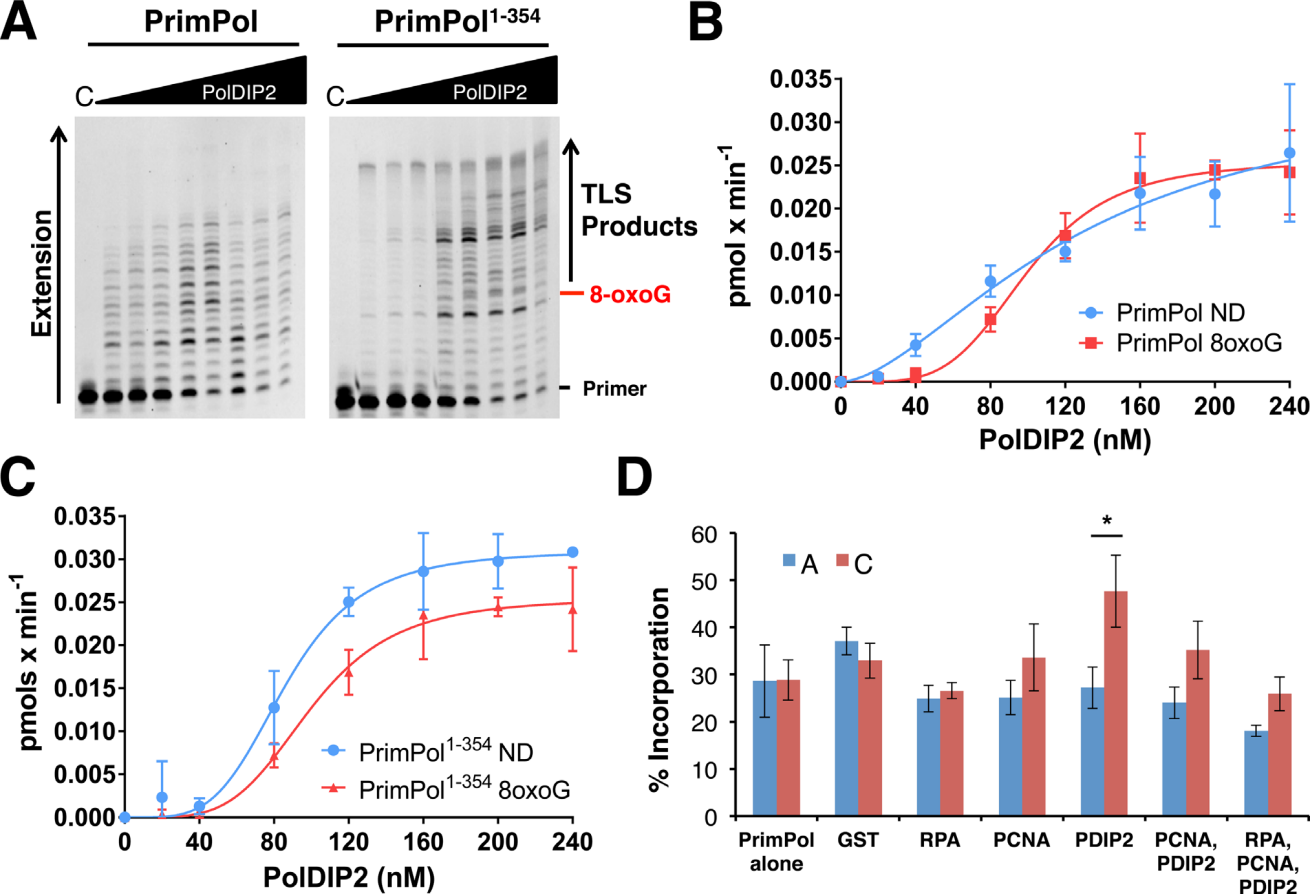
PolDIP2 enhances the efficiency and fidelity of 8-oxoG bypass by PrimPol. (**A**) PrimPol or PrimPol^1–354^ (40 nM) were incubated with dNTPs (100 μM) and 5′-labeled primer/template (20/50-mer) substrates containing a single 8-oxoG lesion 8nt downstream of the primer/template junction in the presence of increasing GST-PolDIP2 concentrations (0, 20, 40, 80, 120, 160, 200, 240 nM) for a single 10 min timepoint. ‘C’ indicates the no enzyme control reaction. (**B**) Relative increase in the rate of TLS product synthesis (as indicated in figure) by PrimPol on non-damaged (ND) and 8-oxoG containing templates in the presence of increasing GST-PolDIP2 concentrations (0, 20, 40, 80, 120, 160, 200, 240 nM). Data were fitted using GraphPad Prism 6 software. Values are the means of four independent experiments. Error bars are ±SD. (**C**) Relative increase in the rate of TLS product synthesis (as indicated on figure) by PrimPol^1–354^ on non-damaged (ND) and 8-oxoG containing templates in the presence of increasing GST-PolDIP2 concentrations (0, 20, 40, 80, 120, 160, 200, 240 nM). Data were fitted using GraphPad Prism 6 software. Values are the means of four independent experiments. Error bars are ±SD. (**D**) PrimPol (100 nM) was incubated with either dATP or dCTP (100μM) and 5′ labeled primer/template (27/50-mer) substrates with a single 8-oxoG lesion as the immediate templating base (position 28 on the template) in the absence and presence of GST-PolDIP2 (300 nM), PCNA (100 nM), and RPA (25 nM), or a combination of each. Reaction products were quantified to give the relative amounts of correct (dCTP, red) and incorrect (dATP, blue) incorporation. Data represent the mean of three independent experiments. Error bars are ±SD. Data were subject to an unpaired t-test, PolDIP2 alone data *P* < 0.05.

PrimPol's 8-oxoG bypass efficiency, in the absence and presence of PolDIP2, was initially investigated using a primer/template (20/50-mer) containing a single 8-oxoG lesion located 8 nucleotides downstream from the primer/template junction (sequences 2 and 10, Supplementary Table S1). We observed that in the presence of increasing PolDIP2 concentrations, PrimPol and PrimPol^1–354^ synthesized a greater number of post-8-oxoG extension products in a dose-dependent manner, compared to reactions containing PrimPol only (Figure 4A–C). However, it is important to note that, unlike Pol λ (16), this stimulation was not significantly greater than that observed on non-damaged DNA templates (Figure 4B and C). Nevertheless, the enhancement of PrimPol's polymerase activity by PolDIP2 does increase the efficiency of 8-oxoG bypass compared to the enzyme alone. Together, these results suggest that PolDIP2 stimulates PrimPol-mediated 8-oxoG bypass by enhancing the polymerase activity of the enzyme rather than the ability of PrimPol to traverse the lesion.

Despite possessing the ability to bypass 8-oxoG lesions, PrimPol's inherent bypass fidelity is relatively poor, displaying around 1:1 error-prone (dATP) to error-free (dCTP) incorporations opposite the 8-oxoG lesion (11). In addition to stimulating 8-oxoG bypass, PolDIP2 has also been shown to increase bypass fidelity by Pols λ and η, but only in the added presence of PCNA and RPA, with the protein alone not affecting lesion bypass fidelity (16). Therefore, we next analyzed the effect of PCNA, RPA, and PolDIP2 on PrimPol's fidelity when bypassing 8-oxoG lesions. To examine this, we employed primer extension assays with a primer/template (27/50-mer), where the immediate templating base was an 8-oxoG lesion (position 28 on the template) (sequences 3 and 10, Supplementary Table S1). Reactions were supplemented with either dATP or dCTP to allow analysis and quantification of error-prone and error-free bypass. As demonstrated previously (11), in the absence of auxiliary proteins PrimPol incorporates both dATP and dCTP opposite the 8-oxoG lesion at ∼ 1:1 ratio (Figure 4D). This ratio was largely unchanged in the presence of RPA, which has previously been shown to increase the fidelity of 8-oxoG bypass by Pol λ and Pol η (32). It should be noted that RPA was used at low concentrations to prevent inhibition of PrimPol (13). PCNA also did not significantly affect the fidelity of lesion bypass by PrimPol. However, in the presence of PolDIP2 alone, PrimPol's fidelity opposite 8-oxoG was significantly improved, with the enzyme demonstrating an almost 2-fold increase in dCTP incorporation, whilst dATP incorporation remained largely the same. However, in the presence of both PolDIP2 and PCNA, or PolDIP2, PCNA, and RPA, in combination, this increase in fidelity was reduced and the overall amount of incorporation (both dATP and dCTP) decreased. These data therefore demonstrate that, unlike Pols λ and η (16), PrimPol's fidelity opposite 8-oxoG is increased in the presence of PolDIP2 alone. Furthermore, addition of RPA and PCNA actually act to lessen the effect of PolDIP2 on PrimPol's lesion bypass fidelity.

The catalytic domain of PrimPol alone has the ability to bypass cyclobutane pyrimidine dimers (CPDs), however the full-length enzyme is stalled by these lesions. In the presence of magnesium, PrimPol is also stalled by abasic (Ap) sites (8,11). We also tested whether PolDIP2 permits bypass of these lesions by PrimPol. In each case, the presence of PolDIP2 did not allow PrimPol to synthesize across the damaged nucleotide (Supplementary Figure S6A). In addition, we analysed the lesion bypass fidelity of PrimPol in the presence of PolDIP2 when traversing a uracil and 6-4PP lesion. Again, PrimPol's fidelity was in line with the previously published results, incorporating dATP opposite uracil, and dTTP opposite the 6-4PP (11).

PrimPol is an error-prone polymerase, which has previously been shown to misincorporate bases and extend base mismatches (13). In particular, the enzyme shows a propensity to misincorporate and extend mismatched bases opposite a templating C (13). Since PolDIP2 increases PrimPol's fidelity when synthesising past an 8-oxoG lesion, we also tested whether the protein affects PrimPol's fidelity on non-damaged DNA. To measure this, we analyzed PrimPol's level of misincorporation opposite a templating C in the absence and presence of PolDIP2. However, we observed that PolDIP2 does not reduce PrimPol's level of misincorporation, suggesting that the protein does not improve PrimPol's fidelity on non-damaged DNA. Together, these results suggest that PolDIP2 acts to increase PrimPol's efficiency and fidelity when specifically bypassing an 8-oxoG lesion, rather than improving the enzymes overall fidelity rates.

### Analysis of the interaction of PolDIP2 with PrimPol

PolDIP2 was originally identified as a potential PrimPol interacting protein in a large-scale pull-down screen performed previously (30). In order to analyse this interaction in more detail, we employed BS_3_ cross-linking and mass spectrometry analysis. This type of analysis allows non-covalent interactions between proteins to be converted into covalent bonds, specifically BS_3_ is able to cross-link primary amines on the side chains of lysine residues, in addition to the N-terminus of proteins. Further protease digestion and MS analysis of cross-linked protein complexes allows the covalent attached regions of each protein to be recognized and thereby interacting regions to be identified (33). StavroX cross-linking analysis software was used to identify and score cross-linked peptides, as detailed previously (29). Since EMSA data suggested an interaction between PolDIP2 and the catalytic domain of PrimPol (Figure 2), untagged PolDIP2 and PrimPol^24–354^ were used for this analysis.

**Figure 5.**
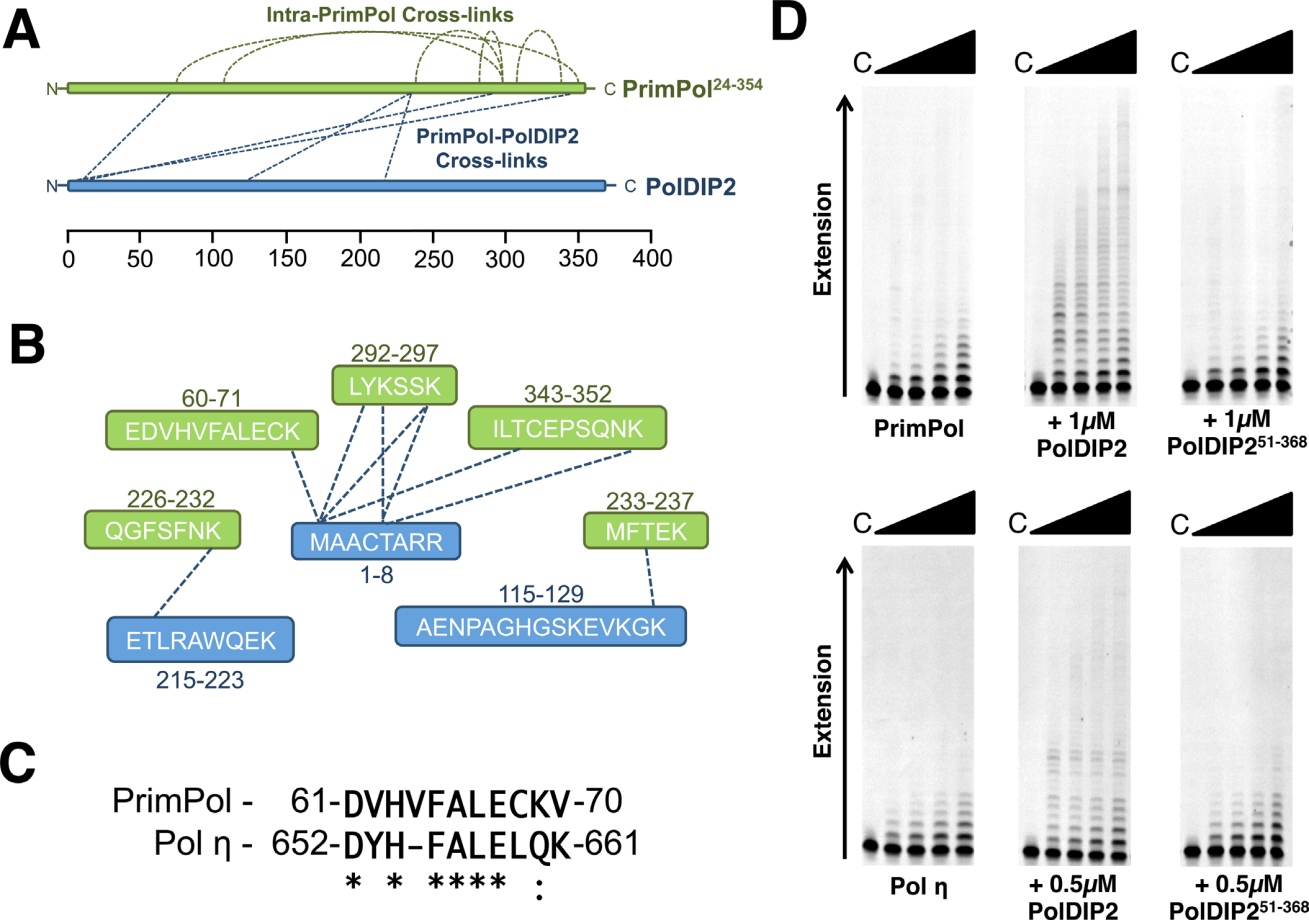
Analysis of the PrimPol-PolDIP2 interactions. (**A**) PrimPol^24–354^ (green) and untagged-PolDIP2 (blue) cross-links were analysed by digest and MS revealing potential interacting regions on each protein (Supplementary Table S2). The locations of intra-PrimPol and inter-PrimPol/PolDIP2 cross-links are indicated by dotted lines. The relative amino acid positions are shown below. (B) The amino acid sequences of PrimPol-PolDIP2 cross-linked peptides. Dotted lines between peptides indicate the specific residues cross-linked in each case. Cross-linked PrimPol peptides are shown in green and PolDIP2 peptides in blue. (**C**) Alignment of the PolDIP2-interacting regions of PrimPol and Pol η showing the high degree of homology between the two peptides. (**D**) PrimPol (100nM) and Pol η were incubated with 5′-labeled primer/template (20/97-mer) substrates and dNTPs (100 μM) in the absence and presence of GST-PolDIP2 or PolDIP2^51–368^. GST-PolDIP2 stimulated the processivity of both PrimPol and Pol η, however PolDIP2^51–368^ failed to stimulate the processivity of either enzyme.

Intriguingly, the vast majority of PrimPol-PolDIP2 cross-links identified were mediated by the N-terminus of PolDIP2 (residues 1–8), with additional secondary crosslinks also identified (Supplementary Table S2, Figure 5A and B). The most abundant and highest scoring cross-linked peptide identified on PrimPol, which cross-linked to the N-terminus of PolDIP2, was located between amino acid positions 60 and 70 on the full-length protein (EDVHVFALECK), with the cross-linked residue identified as lysine 70 (Supplementary Table S2). Notably, this peptide displays strong homology to a region of Pol η previously found to mediate the enzyme's interaction with PolDIP2 (Figure 5C) (15), potentially suggesting that PrimPol and Pol η share a similar mode of binding to PolDIP2. A number of other cross-linked peptides were also identified on PrimPol. The majority of these were located towards the C-terminus of the truncated protein. However, analysis of intra-PrimPol cross-links, suggests that these regions are in close proximity to the EDVHVFALECK peptide in the folded protein (Supplementary Table S3, Figure 5A and B).

Given that the N-terminal 50 amino acids of PolDIP2 comprise a mitochondrial targeting sequence (MTS), which is likely cleaved off upon entry to the mitochondria (19), it was somewhat surprising to identify this region as the mediator of the PrimPol interaction. In order to validate the findings of the crosslinking and MS analysis, we generated a truncated form of PolDIP2 lacking the first 50 amino acids (PolDIP2^51–368^) and assayed its ability to stimulate PrimPol's processivity in comparison to the full-length protein. In addition, we also analysed the effect of PolDIP2^51–368^ on the processivity of Pol η. In line with previous results, we find that full-length PolDIP2 is able to stimulate the processivity of both PrimPol and Pol η (Figure 5D) (16). However, in contrast, PolDIP2^51–368^ failed to stimulate the processivity of either enzyme. This assay was repeated across a range of PolDIP2^51–368^ concentrations with no increase in PrimPol's processivity identified (Supplementary Figure S8A). Furthermore, PolDIP2^51–368^ failed to produce an increase in the DNA binding of PrimPol, which was previously observed with the full-length protein (Supplementary Figure S8B). These results further support the findings of the MS analysis, suggesting that the interaction between PolDIP2 and PrimPol is mediated by the N-terminus of PolDIP2. Furthermore, these findings suggest that Pol η may also interact with the N-terminus of PolDIP2.

### Depletion of PolDIP2 causes slowed replication fork rates after UV damage

Despite the inability of PolDIP2^51–368^ to stimulate the processivity of Pol η, existing data suggests that these proteins do share a functional interaction *in vivo*. Specifically, it has been shown that depletion of PolDIP2 causes persistent Pol η foci in the absence of damage. Furthermore, PolDIP2 depleted cells showed increased UV sensitivity to a similar level as cells lacking Pol η (XPV cells) however, no further increase in sensitivity was observed when PolDIP2 was depleted in XPV cells (15). Additionally, cells depleted of PolDIP2 showed an increased sensitivity to oxidative damage (16). These studies implicate PolDIP2 in the regulation of TLS *in vivo*, although the direct impact of depletion of PolDIP2 on DNA replication has not previously been examined.

**Figure 6.**
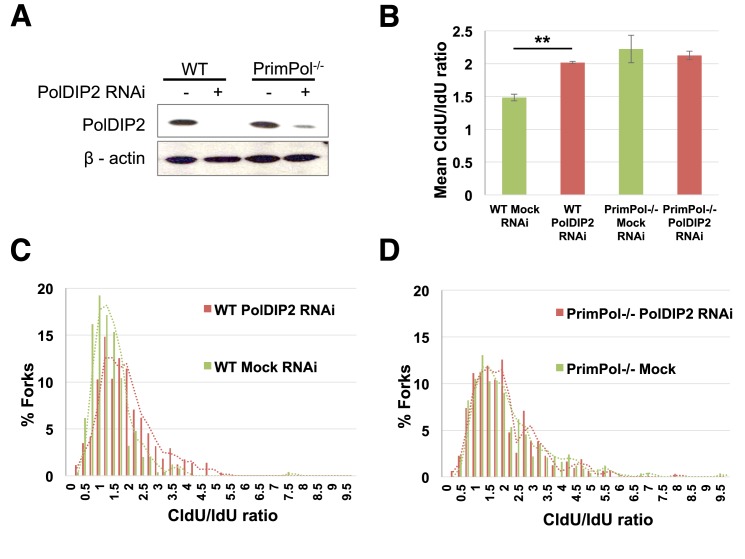
Depletion of PolDIP2 causes decreased replication fork rates following UV damage. (**A**) Western blot analysis of PolDIP2 depletion by siRNA in wild-type (WT) and PrimPol^−/−^ MRC5 cells, compared to mock depleted cells. (**B**) DNA replication fork rates in PolDIP2 siRNA or mock treated wild-type and PrimPol^−/−^ MRC5 cells were analysed by DNA fibre analysis. Cells were pulsed with CldU for 20 mins followed by UV irradiation (20 J/m^2^) and pulse labelled again with IdU for a further 20 min. The mean CldU/IdU ratio of wild-type and PrimPol^−/−^ MRC5 cells either mock (green) or PolDIP2 siRNA (red) treated is shown. Error bars indicate ±SE (*n* = 3). Data were subject to an unpaired *t*-test, showing a significant difference in mean CldU/IdU ratio between the mock and PolDIP2 RNAi treated wild-type MRC5 cells (*P* < 0.01), but not between the PrimPol^−/−^ cells. (**C**) DNA replication fork rate data for wild-type MRC5 cells shown as the ratio of CldU to IdU (*n* = 3). Green lines indicate analysis of mock treated cells, whilst red lines indicated analysis of PolDIP2 siRNA treated cells. (D) DNA replication fork rate data for PrimPol^−/−^ MRC5 cells shown as the ratio of CldU to IdU (*n* = 3). Green lines indicate analysis of mock treated cells, whilst red lines indicated analysis of PolDIP2 siRNA treated cells.

To analyse the impact of depletion of PolDIP2 on replication fork rates following DNA damage, both wild-type and PrimPol^−/−^ MRC5 cells were either PolDIP2 siRNA or mock treated before DNA fibre analysis was conducted (Figure 6A). Cells were pulse labelled with chlorodeoxyuridine (CldU) for 20 min before UV irradiation (20 J/m^2^), following this, cells were pulse labelled again with iododeoxyuridine (IdU) for an additional 20 min and the ratios of the two labels determined. Significantly, depletion of PolDIP2 in wild-type MRC5 cells causes a significant decrease in replication fork rates following UV-C irradiation (Figure 6B–D). This suggests that PolDIP2 is involved in DNA replication and, more specifically, in DNA damage tolerance, supporting published studies implicating it in TLS processes. Although to a lesser extent than observed in the previously studied PrimPol^−/−^ DT40 cells (8), MRC5 cells lacking PrimPol also display a decrease in replication fork rates following UV-C irradiation. However, intriguingly, depletion of PolDIP2 in PrimPol^−/−^ cells did not produce a further decrease in replication fork rates, suggesting that PrimPol and PolDIP2 work epistatically in the same pathway to promote continued DNA replication in the presence of UV damage (Figure 6B–D). This also suggests that PolDIP2 may also operate in a post-replicative manner during gap-filling by other TLS polymerases, potentially explaining why a further decrease in replication fork rates was not observed, despite PolDIP2 likely partnering other TLS enzymes.

## DISCUSSION

Previous studies have implicated PolDIP2 in TLS damage tolerance processes through the regulation of Pols λ and η (15,16). In addition, this protein has also been shown to interact with PCNA, Pol δ, Pol ζ and Rev1 (15). In this current study, we show that PolDIP2 is also involved in the regulation of PrimPol's polymerase activity. Specifically, PolDIP2 is able to increase the polymerase activity of PrimPol by increasing the enzyme's DNA binding capacity and processivity. In addition, PolDIP2 also acts as a fidelity factor for PrimPol during the bypass of 8-oxoG, enhancing dCTP incorporation opposite this oxidative lesion. In contrast, PolDIP2 has a minimal effect on PrimPol's primase activity. This suggests that the protein acts specifically to promote PrimPol's polymerase activity. Previously, it has been shown that PrimPol's ZnF domain is required for the primase activity of the enzyme, and additionally, is involved in negatively regulating its processivity (11). This raises the possibility that binding of PolDIP2 may alleviate this negative regulation by the ZnF domain, in turn promoting increased processivity of the enzyme. Together, the *in vitro* data presented here suggests that PolDIP2 increases the processivity and polymerisation rates of PrimPol by stabilising the enzyme on DNA and improving PrimPol's inherently poor DNA binding capacity (11).

Previously published results, and data presented here, suggest that PrimPol is not regulated through a canonical PCNA-mediated polymerase switch mechanism (13). Furthermore, the presence of PCNA inhibited the positive impact of PolDIP2 on PrimPol's primer extension activity. It has previously been suggested that PolDIP2 might act as a bridging factor to enhance polymerase-PCNA interactions and thereby further stimulate polymerase activity (16). However, our data imply that PolDIP2 acts alone, in the absence of PCNA, to enhance PrimPol's activity. In further support of this, it was previously found that PolDIP2 only enhances Pol η and λ bypass fidelity opposite 8-oxoG bypass in the presence of RPA and PCNA (16). However, in the case of PrimPol, we observed that PolDIP2 alone is sufficient to increase its 8-oxoG bypass fidelity and the further presence of RPA and PCNA actually reduces this effect.

Additionally, we found that PrimPol possesses a potential PolDIP2 binding motif with homology to that of Pol η (15). Interestingly, we identified that this motif appears to bind to the very N-terminus of PolDIP2. However, the first ∼50 amino acids of PolDIP2 are thought to comprise a MTS and are likely cleaved off upon entry to the mitochondria (19). PolDIP2 lacking this MTS was not able to stimulate the processivity of either PrimPol or Pol η. Importantly, previous studies reporting stimulation of Pols η, λ and δ by PolDIP2 only employed full-length PolDIP2 with the N-terminal 50 amino acids intact (16). Additionally, it was originally reported that PolDIP2 inhibits Pol δ activity at higher concentrations, however this study was performed using truncated PolDIP2 without the MTS (19). Therefore, it seems that these contradictory reports can be explained by the different PolDIP2 constructs used in each case. Furthermore, these reports support data presented here that the first 50 amino acids of PolDIP2 are required for stimulation of, and likely the interaction with, polymerases including not only PrimPol but also Pol η and Pol δ.

Despite this, *in vivo* data does support a role for PolDIP2 in DNA replication, and more specifically the regulation of TLS. Here, we have shown that depletion of PolDIP2 from cells causes a decrease in replication fork rates following UV irradiation to a similar level as that seen with PrimPol^−/−^ cells. Furthermore, depletion of PolDIP2 in PrimPol^−/−^ cells does not produce a further decrease in fork speed. Therefore, it appears that PolDIP2 and PrimPol cooperate to promote continued replication in the presence of DNA damage. Importantly, this does not rule out the possibility that PolDIP2 also assists other TLS polymerases in a post-replicative gap filling manner. Indeed, this is supported by previous studies suggesting that PolDIP2 acts to promote interactions between canonical TLS polymerases and PCNA. In support of this, previous reports suggest that PCNA ubiquitination is not required to maintain normal fork progression on damaged DNA but is instead essential for filling-in post-replicative gaps (34).

Importantly, initial characterisation of PolDIP2 suggested that the protein was primarily mitochondrial (19). However, it was also acknowledged that PolDIP2 may also be present in the nucleus in small amounts and that interactions between PolDIP2 and Pol δ may only occur during specific cellular events, such as following DNA damage. This study also suggested that additional isoforms of PolDIP2 may exist, which localizes to the nucleus and possibly serves different functions to those in the mitochondria (19). Since this initial characterisation, additional reports indicate that PolDIP2 does indeed localize to the nucleus, with an increase following UV damage (20). Therefore, it is possible that nuclear and mitochondrial PolDIP2 fulfil different functions *in vivo*. Whilst PolDIP2 localised to the mitochondria will likely have its MTS removed, it is possible that a small amount of PolDIP2, which localizes to the nucleus does not. This suggests that the stimulatory effects of PolDIP2 on PrimPol may be primarily nuclear and in response to DNA damage, rather than mitochondrial. PrimPol is a highly error-prone enzyme and must be strictly regulated to restrict its contribution to DNA synthesis (13). Thus, it seems likely that the PrimPol-PolDIP2 interaction may also be mediated by post-translational modifications in response to DNA damage, this would prevent PrimPol's DNA binding and processivity from being constantly enhanced and therefore limit its involvement in DNA synthesis. However, further studies are required to assess potential PrimPol and PolDIP2 post-translational modifications and their influence on the interactions between these proteins.

Overall, the findings presented here establish that PolDIP2 is able to enhance the polymerase activities of PrimPol *in vitro*. In support of these findings, we also demonstrate that cells depleted of PolDIP2 show replication defects similar to PrimPol^−/−^ cells after UV irradiation. These effects are not further increased when PolDIP2 is depleted in PrimPol^−/−^ cells, clearly suggesting that PolDIP2 is a binding partner of PrimPol *in vivo* and likely mediates its TLS and primer extension activities in response to DNA damage. These findings further support the accumulating published evidence implicating PolDIP2 as a general DNA damage tolerance factor involved in promoting TLS by a number of different polymerases. Together, our work describes a new regulatory partner of PrimPol and enhances our understanding of the emerging role of PolDIP2 in TLS.

## Supplementary Material

SUPPLEMENTARY DATA
