# Supplementary material for: PolDIP2 interacts with human PrimPol and enhances its DNA polymerase activities
Source: Nucleic Acids Res. 2016 Mar 16;44(7):3317–29. doi: 10.1093/nar/gkw175 (PMC4838387; doi:10.1093/nar/gkw175)
Supplement: SUPPLEMENTARY DATA [file supp_44_7_3317__index.html]

PolDIP2 interacts with human PrimPol and enhances its DNA polymerase activities — PolDIP2 interacts with human PrimPol and enhances its DNA polymerase activities — SUPPLEMENTARY DATA 

# PolDIP2 interacts with human PrimPol and enhances its DNA polymerase activities

## SUPPLEMENTARY DATA

- SUPPLEMENTARY DATA
