# Supplementary material for: PolDIP2 interacts with human PrimPol and enhances its DNA polymerase activities
Source: Nucleic Acids Res. 2016 Mar 16;44(7):3317–29. doi: 10.1093/nar/gkw175 (PMC4838387; doi:10.1093/nar/gkw175)
Supplement: SUPPLEMENTARY DATA [file supp_gkw175_nar-03059-d-2015-File008.pdf]

Supplementary Tables

Table S1: Sequences of the DNA oligonucleotides used in the primase and primer extension assays. Lesions within the sequences are denoted in red.

Table S2. MS analysis of PolDIP2-PrimPol cross-linked peptides. The amino acid sequence of cross-linked PolDIP2 (green) and PrimPol (blue) peptides identified in the MS analysis are shown. Crosslinked residues in each case are shown in bold. Peptide location indicates the location of peptide in the specific constructs used for the analysis, not the wild-type protein. Scores are based on the probability of the occurrence of an ion in addition to the intensity of the signal in the fragment spectrum, as given by the StavroX analysis software. 'm/z' indicates the mass to charge ratio, 'z' shows the charge of the precursor.

Table S3. MS analysis of intra-PrimPol cross-links. The amino acid sequence of cross-linked PrimPol (blue) peptides identified in the MS analysis are shown. Cross-linked residues in each case are shown in bold. Peptide location indicates the location of peptide in the specific constructs used for the analysis, not the wild-type protein. Scores are based on the probability of the occurrence of an ion in addition to the intensity of the signal in the fragment spectrum, as given by the StavroX analysis software. 'm/z' indicates the mass to charge ratio, 'z' shows the charge of the precursor.

#	Oligonucleotide	Label	Sequence
1	HP-16 Primer	5'-Hex	5′-CACTGACTGTATGATG-3′
2	HP-20 Primer	5'-Hex	5′-TGTCGTCTGTTCGGTCGTTC-3′
3	HP-27 Primer	5'-Hex	5′-TGTCGTCTGTTCGGTCGTTCGGTCTTC-3′
4	ND-50 $Template_{TT}$	None	5′-CGCGCAGGGCGCACAACAGCCTTGAAGACCGAACGACCGAACAGACGACA-3′
5	ND-50 $Template_{CC}$	None	5′-CGCGCAGGGCGCACAACAGAGCCGAAGACCGAACGACCGAACAGACGACA-3′
6	ND-97 Template	3'-Biotin	5′- ACCGCGAACTTGAATTCTAGTTCAGTCTAAATGCTCTCAAGCACTGAGCAATTCACAACATATGGCTTT CGATTACCGAACGACCGAACAGACGACA-3′
7	Primase Template	5'-Biotin	5′-GTCTTCTATCTCGTCTATATTCTATTGTCTCTATGAATACCTTCATCAGTCTCACATAGATGCATC-3′
8	6-4(PP) Template	None	5′- CTCGTCAGCATCT^TCATCATACAGTCAGTG-3′
9	CPD Template	None	5′-CGCGCAGGGCGCACAACAGCCT=TGAAGACCGAACGACCGAACAGACGACA-3′
10	8-oxo-G Template	None	5′-CGCGCAGGGCGCACAACAGCC8-oxo-GTGAAGACCGAACGACCGAACAGACGACA-3′
11	dUracil Template	None	5′-CGCGCAGGGCGCACAACAGCCUTGAAGACCGAACGACCGAACAGACGACA-3′
12	AP Template	None	5′-CGCGCAGGGCGCACAACAGCCAPTGAAGACCGAACGACCGAACAGACGACA-3′

Table S1

Score	m/z	z	Measured Mass	Calculated Mass	PolDIP2 Peptide	Peptide location	PrimPol Peptide	Peptide location
131	807.389	3	2420.153	2420.152	**M**AABTARR	0-8	EDVHVFALEB**K**	58-68
105	411.462	4	1642.827	1642.829	MAAB**T**AR	0-7	LYKSS**K**	290-295
101	755.038	3	2263.099	2263.099	**M**AABTARR	0-8	IL**T**BEPSQNK	341-350
90	566.53	4	2263.099	2263.099	MAAB**T**ARR	0-8	ILTBEPSQN**K**	341-350
87	767.721	3	2301.149	2301.154	AENPAGHGSKEVKG**K**	115-129	MF**T**EK	231-235
86	411.462	4	1642.827	1642.829	MAAB**T**AR	0-7	LY**K**SSK	290-295
84	532.022	4	2125.065	2125.071	E**T**LRAWQEK	215-223	QGFSFN**K**	224-230
81	709.027	3	2125.065	2125.071	E**T**LRAWQEK	215-223	QGFSFN**K**	223-230
63	1132.054	2	2263.101	2263.099	**M**AABTARR	0-8	IL**T**BEPSQNK	341-350
43	548.28	3	1642.825	1642.829	**M**AABTAR	0-7	L**Y**KSSK	290-295
40	553.612	3	1658.821	1658.824	**M**AABTAR	0-7	LYKSS**K**	290-295
32	566.53	4	2263.098	2263.099	MAAB**T**ARR	0-8	ILTBEPSQN**K**	341-350
29	411.462	4	1642.826	1642.829	MAAB**T**AR	0-7	LY**K**SSK	290-295

Table S2

Score	m/z	z	Measured Mass	Calculated Mass	Peptide 1	Peptide location	Peptide 2	Peptide location
172	665.01	3	1993.015	1993.012	F**S**DTLR	335-340	VALEVTEDN**K**	300-309
137	712.013	3	2134.025	2134.031	Q**K**	351-353	SB**K**EDVHVFALE BK	55-68
30	496.439	5	2478.165	2478.17	**S**SK	293-295	mFTE**K**ATEESW TSNSKK	231-247
27	541.628	6	3244.729	3244.725	NFRLYK**S**SK	287-295	VALEVTEDNKFF PIQS**K**	300-316
25	1311.319	3	3931.943	3931.935	PANPGADGK**K**	118-127	DVSDEYQYFLSS LVSNVRFSD**T**LR	317-340
20	578.709	5	2889.514	2889.515	IG**K**R	296-299	NNmGE**K**HLFVD LGVYTRNR	268-286
15	1475.068	3	4423.189	4423.19	IYLVTTYAEFWFYY**K**	75-89	NNmGEKHLFVD LGVY**T**RNR	268-286
11	712.013	3	2134.025	2134.031	Q**K**	351-353	SB**K**EDVHVFALE BK	55-68

Table S3

Supplementary Figures

Fig. S1. PolDIP2 stimulates full-length primer extension by PrimPol. PrimPol (100nM) was incubated with 5' labelled 20/50-mer primer/template substrates (20nM) and dNTPs (100µM) in the absence or presence of increasing concentrations of GST-PolDIP2 over a time course (1, 3, 5, 10, 20 mins). 'C' indicates the no enzyme control. Quantification of the data is shown in Fig. 1 C and D.

Fig. S2. PolDIP2 does not increase primer synthesis by PrimPol.

(A) The primase activity of PrimPol was analysed in the absence and presence of GST-PolDIP2 or GST only over a range of concentrations (as indicated).

(B) Quantification of the data shown in A, showing the percentage of primer synthesis reaction products generated in the presence of varying concentrations of GST-PolDIP2 or GST only. Nucleotide size markers are shown on the left of the figure.

Fig. S3. Untagged PolDIP2 increases PrimPol's processivity at higher concentrations. Untagged-PolDIP2 was titrated into reactions containing PrimPol (100nM) and 5' labelled 20/97-mer primer/template substrates (20nM). Reactions were initiated with dNTPs (100µM) and excess trap DNA and quenched at 0.5, 1, and 2 min time-points.

Fig. S4. PolDIP2 does not allow PrimPol to displace mtSSB or RPA. The primer extension activity of PrimPol was analysed in the absence and presence of PolDIP2 and mtSSB or RPA using a 5' labelled 20/97-mer primer/template substrate. 'C' indicates the no enzyme control.

Fig. S5. PrimPol is inhibited in the presence of PolDIP2 and PCNA in combination. PrimPol's (100nM) polymerase activity was examined using a 5' labelled 20/97-mer primer/template substrate in the presence of PCNA alone, or a combination of PCNA and PolDIP2 over a time course (1, 3, 5, 10, 20 mins). 'C' indicates the no enzyme control.

Fig. S6. PolDIP2 does not enable PrimPol to bypass CPDs or Ap sites, or alter the fidelity of dU or 6-4pp bypass.

(A) PrimPol was incubated with dNTPs (100μM) and 5'-labelled 20/50-mer primer/template substrates containing a single CPD or Ap site 8nt downstream of the primer-template junction in the absence or presence of PolDIP2 (800nM). Reactions were monitored over a time-course of 1, 3, 5, 10, and 20 mins. 'C' indicates the no enzyme control reaction.

(B) PrimPol was incubated with single dNTPs (dATP, dCTP, dGTP, or dTTP) (100μM) and 5'labelled primer/template substrates with a single dU or 6-4pp as the immediate templating base in the presence of GST-PolDIP2. 'C' indicates the no dNTP control.

Fig. S7. PolDIP2 does not affect PrimPol's fidelity on non-damaged DNA.

(A) PrimPol was incubated with single dNTPs (dATP, dCTP, dGTP, or dTTP) (100μM) and 5'-labelled 27/50-mer primer/template substrates containing dC as the immediate templating base. Reactions were monitored over a time-course of 1, 3, 5, 10, and 20 mins. 'C' indicates the no dNTP control reaction.

(B) The data shown in A were normalised against the correct incoming base (dGTP) and quantified to give the relative misincorporation of each base opposite the templating dC.

Fig. S8. PolDIP2$^{51\text{-}368}$ does not stimulate PrimPol's processivity or DNA binding.

(A) PolDIP2$^{51\text{-}368}$ was titrated into reactions containing PrimPol (100nM) and 5' labelled 20/97-mer primer/template substrates (20nM). Reactions were initiated with dNTPs (100μM) and excess trap DNA and quenched at 0.5, 1, 2, 5, and 10 min time-points.

(B) PrimPol$^{24\text{-}354}$ was incubated with increasing concentrations of PolDIP2$^{51\text{-}368}$ ((0.05, 0.1, 0.2, 0.3, 0.4, 0.5, 0.75, 1 μM)) in EMSA reactions containing 5'-labelled 20/97-mer primer-template substrates.

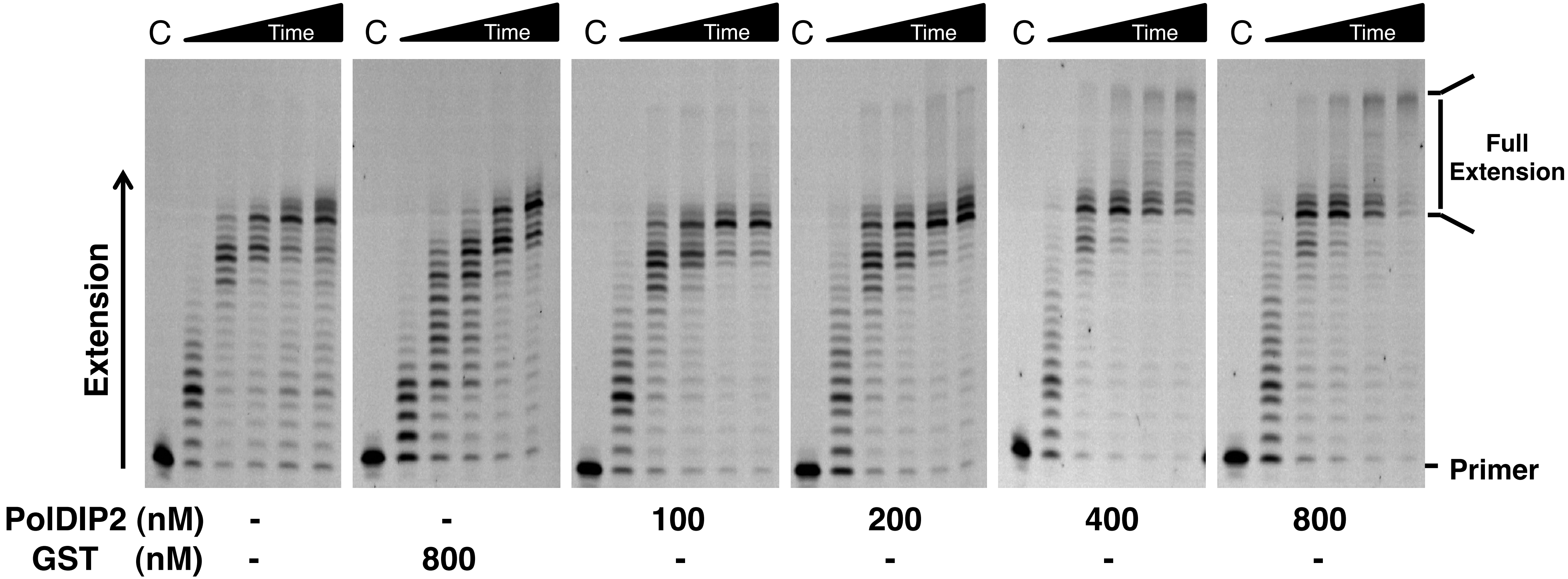
C
Time
Extension
Full Extension
Primer
PolDIP2 (nM)
-
-
100
200
400
800
GST (nM)
-
800
-
-
-
-

Figure S1

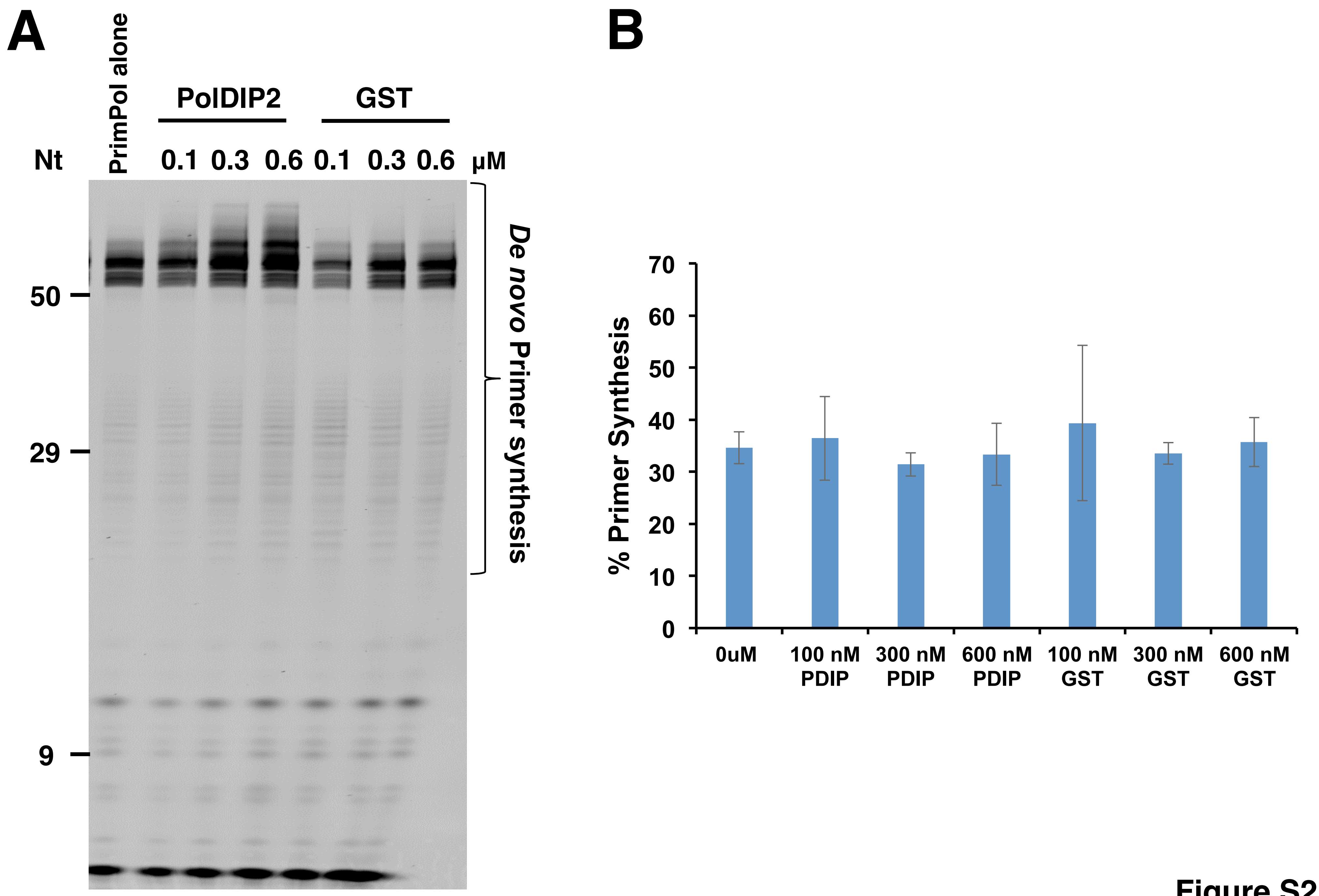
A
PrimPol alone
PolDIP2
GST
Nt
0.1 0.3 0.6 0.1 0.3 0.6 µM
50
29
9
De novo Primer synthesis
B
% Primer Synthesis
70
60
50
40
30
20
10
0
0uM
100 nM PDIP
300 nM PDIP
600 nM PDIP
100 nM GST
300 nM GST
600 nM GST

Figure S2

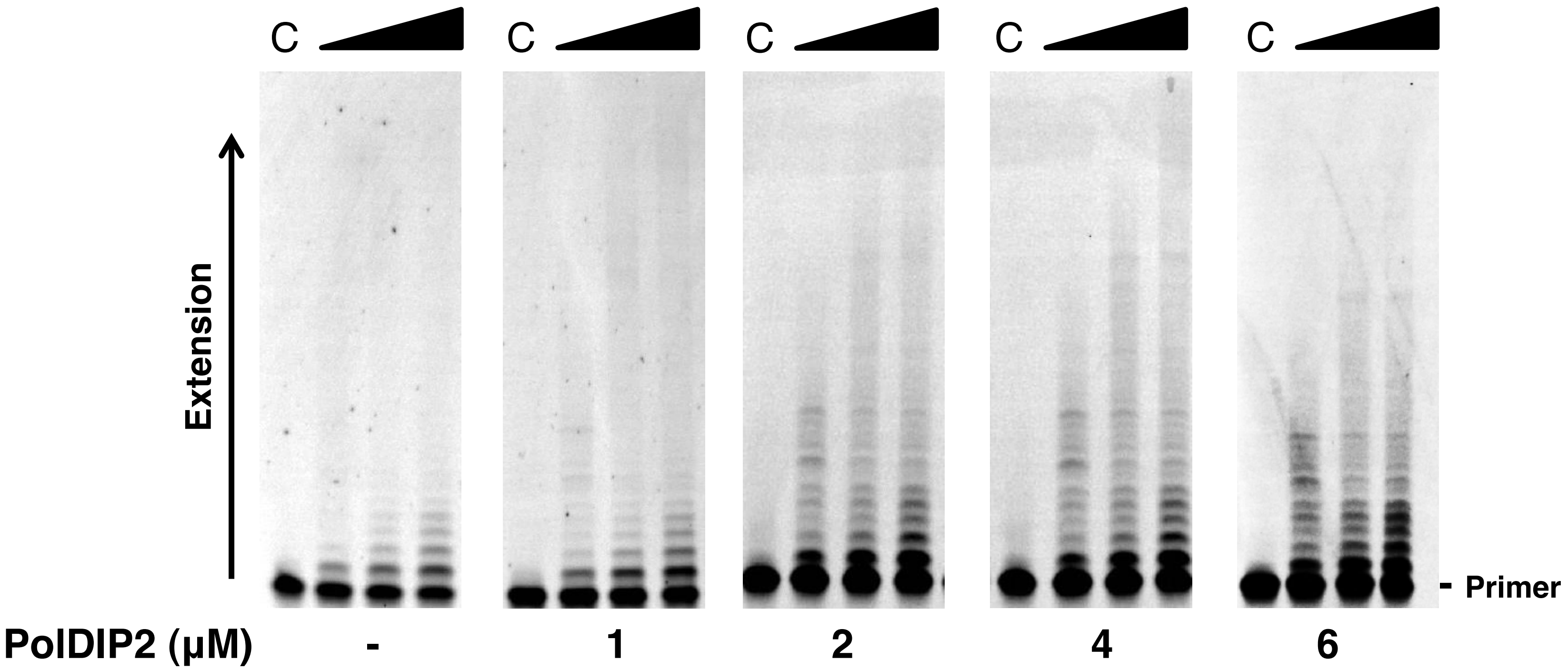

Figure S3

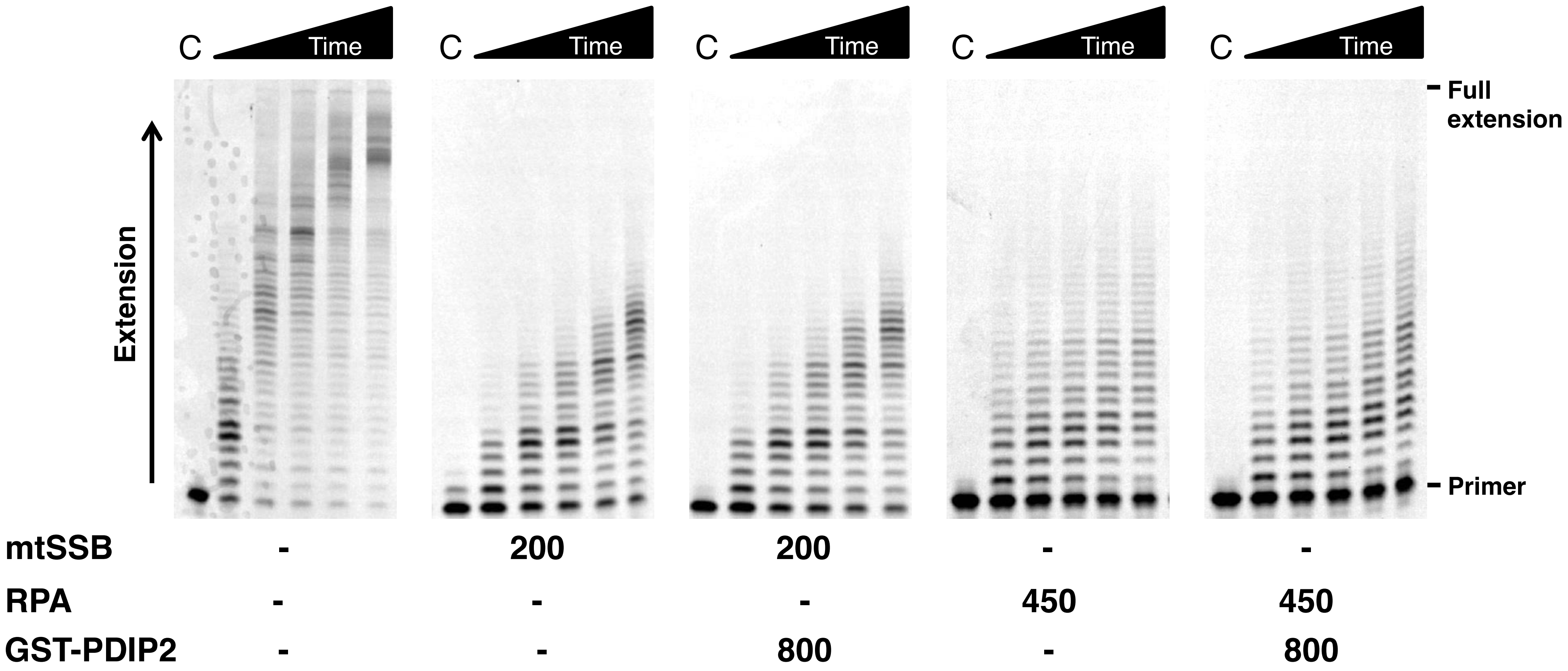
C
Time
Extension
Full extension
Primer
mtSSB - 200 200 - -
RPA - - - 450 450
GST-PDIP2 - - 800 - 800

Figure S4

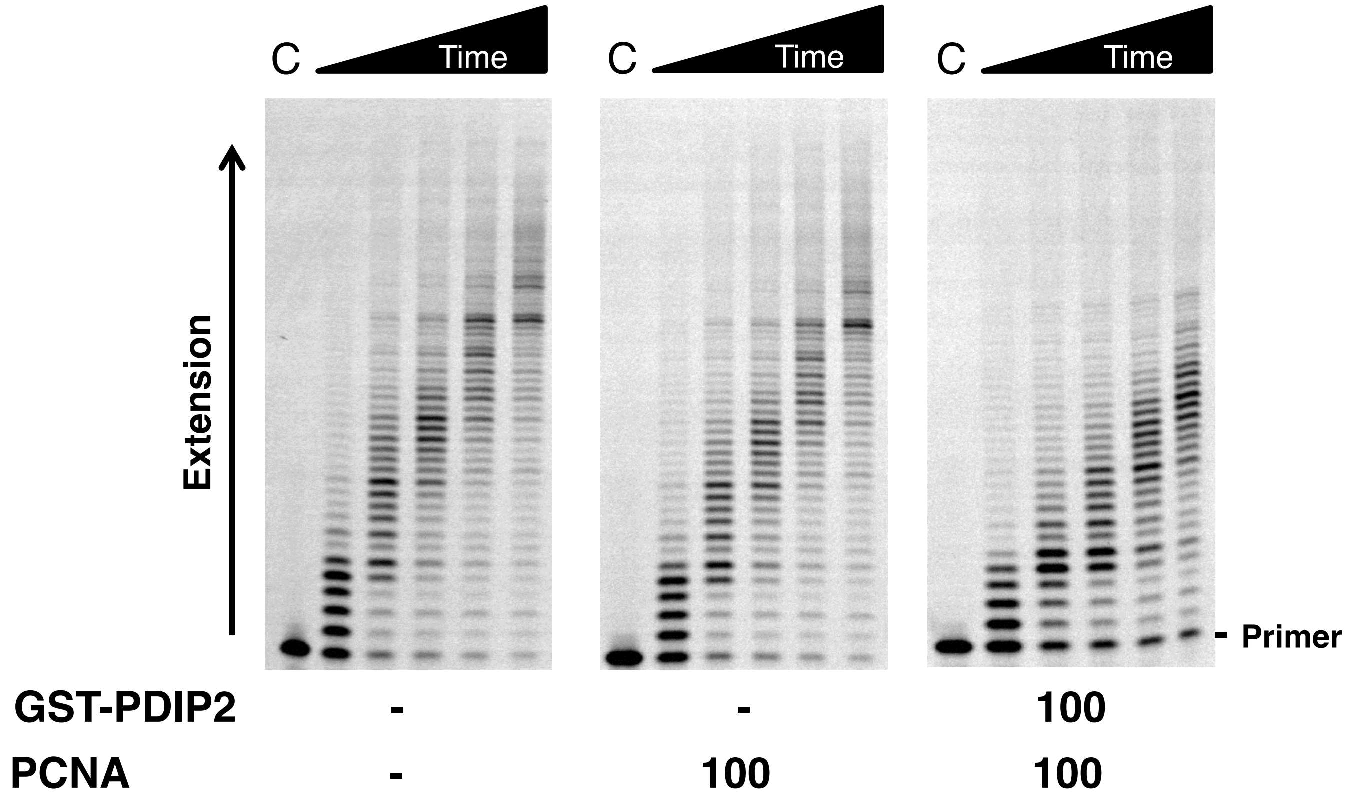
C
Time
C
Time
C
Time
Extension
Primer
GST-PDIP2
-
-
100
PCNA
-
100
100

Figure S5

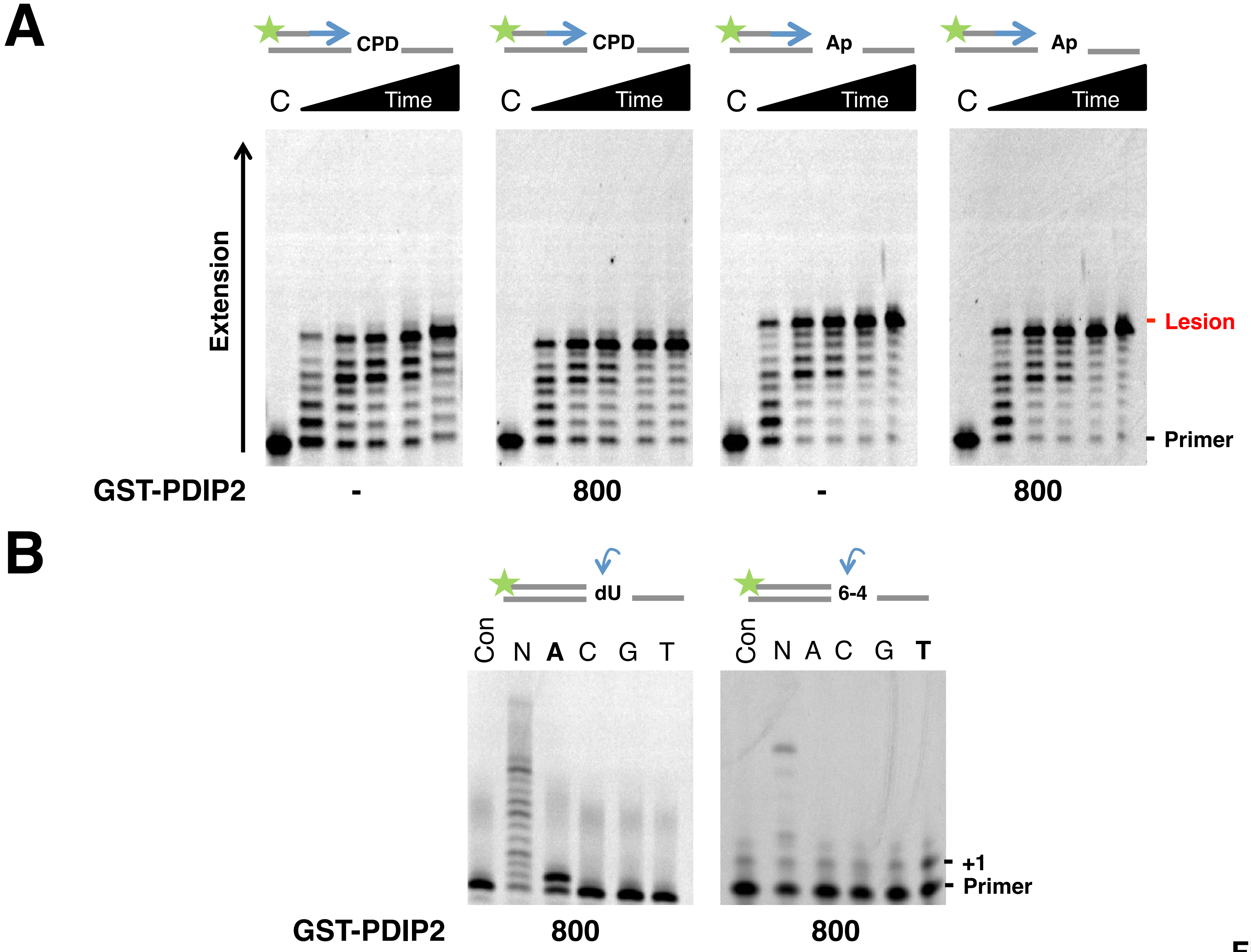
A
CPD
CPD
Ap
Ap
C
Time
Extension
Lesion
Primer
GST-PDIP2
-
800
-
800
B
dU
6-4
Con
N
A
C
G
T
+1
Primer
GST-PDIP2
800
800

Figure S6

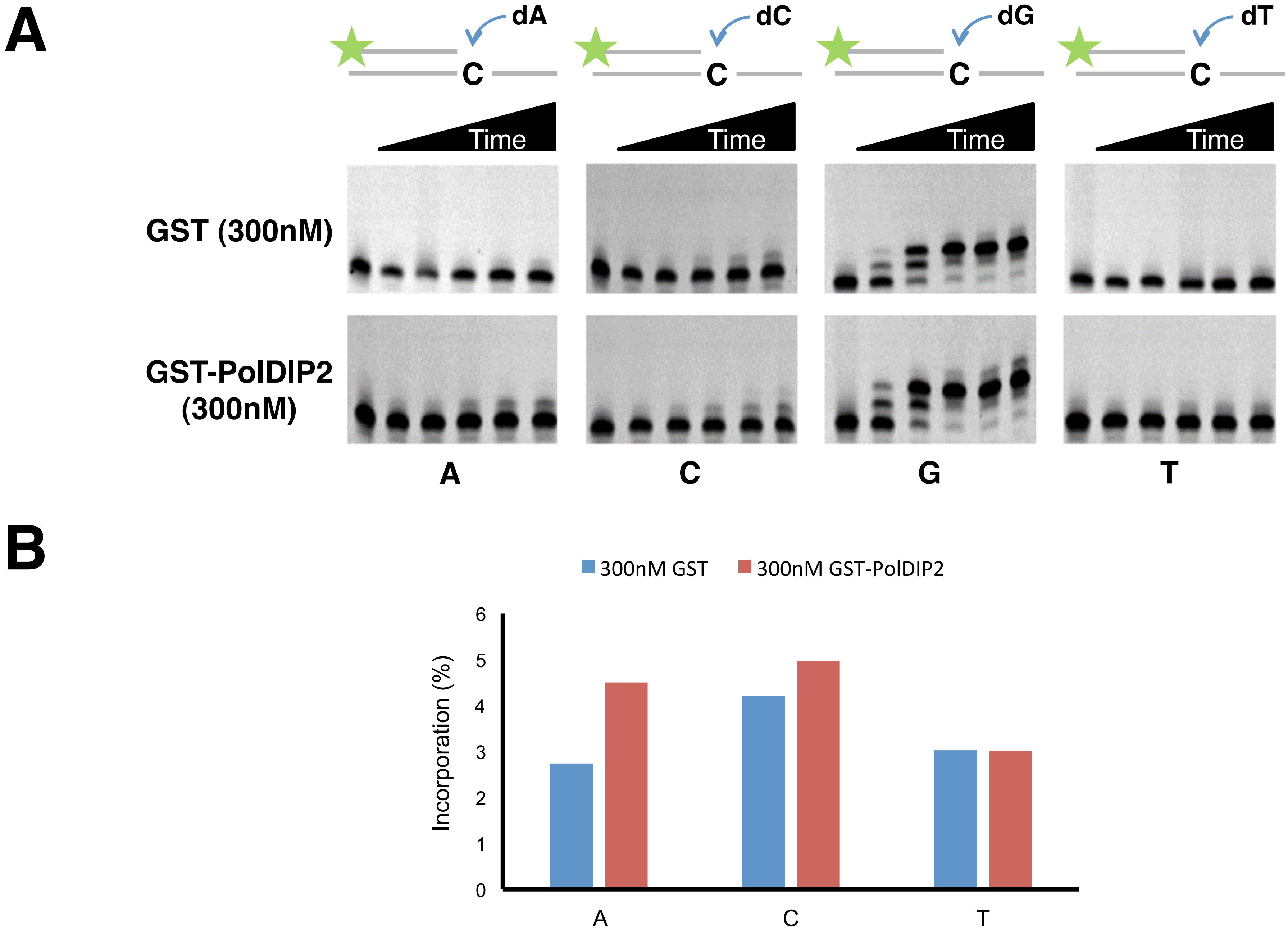
A
dA
dC
dG
dT
C
Time
GST (300nM)
GST-PolDIP2 (300nM)
A
C
G
T
B
300nM GST
300nM GST-PolDIP2
Incorporation (%)
0
1
2
3
4
5
6
A
C
T

Figure S7

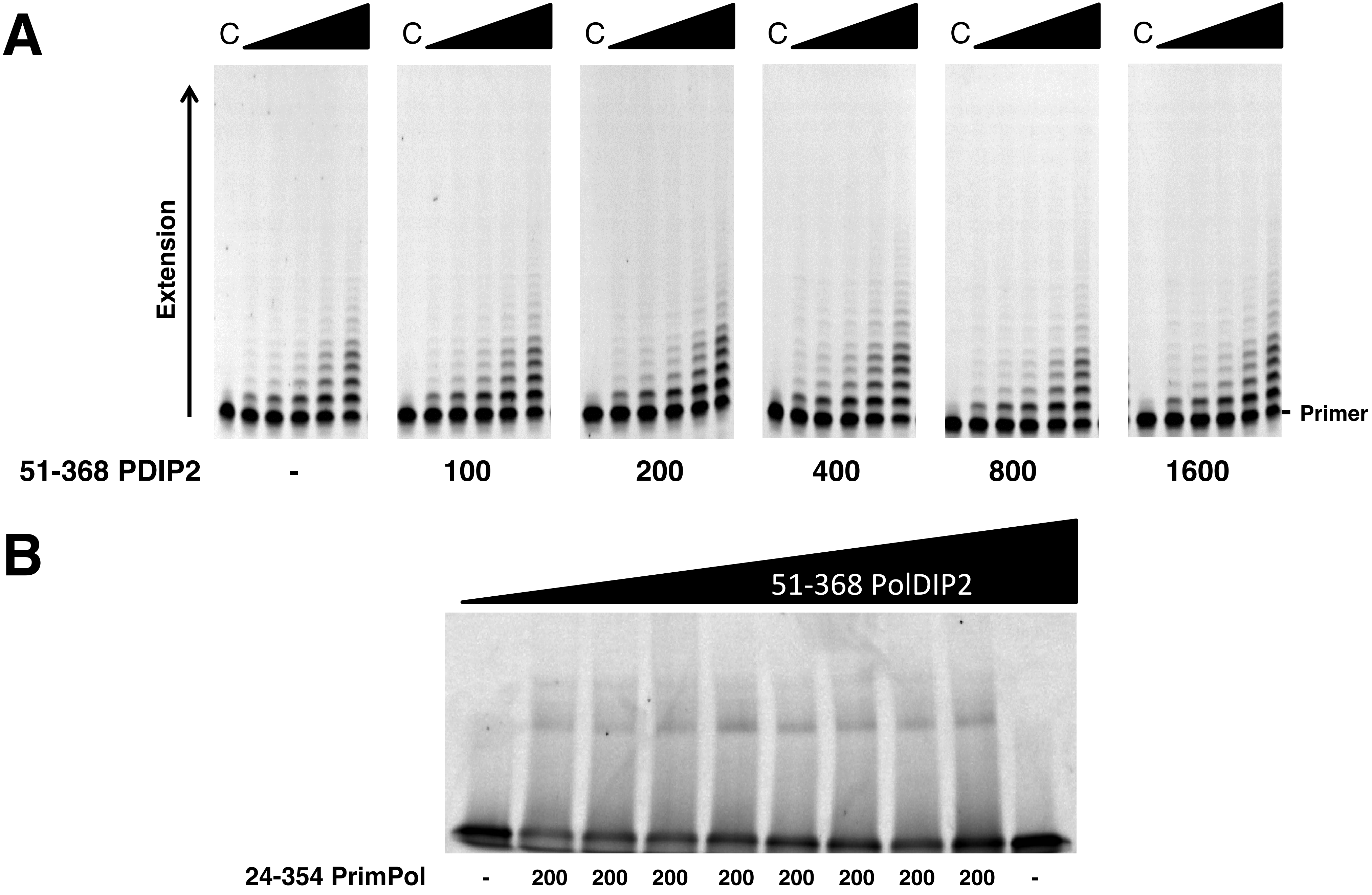
A
C
C
C
C
C
C
Extension
Primer
51-368 PDIP2
-
100
200
400
800
1600
B
51-368 PolDIP2
24-354 PrimPol
-
200
200
200
200
200
200
200
200
-

Figure S8
